# Hasty Conclusions

**Published:** 1905-07

**Authors:** 


### Editorial.

### HASTY CONCLUSIONS.

The literature of dentistry is replete with crude decisions based on incomplete examinations and equally erroneous deductions from faulty research work. It is not to be expected that the earnest historical writer, ever seeking for corroborative details, will be developed speedily in our young profession. This exactness comes only from matured thinking and generations of experience. It must, however, be regarded as true that dentistry is inclined to run to hasty conclusions more extensively than is warranted even through youthful extravagant expression.

This is largely due, it is thought, to careless habits in writing. The essayist who aims to prepare a paper for a society sits down to the task apparently, in many instances, to develop this from memory and experience. The memory side will be prepared from quotations copied from the last article read on a related subject, and no effort will be made to establish the truth of statements or to correct errors of history. This leads to false records, and false history in dentistry is becoming more and more a source of annoyance, and will result in the future to the great injury of the truly original toilers in the profession. The plagiarist we will probably always have with us, but his first cousin, the careless writer, ought to be eliminated by individual effort. The first is the manifestation of degeneracy, the latter of pure physical inertia.

The writer could multiply instances of this peculiarity, but this does not seem necessary in view of the fact that it is constantly made apparent in books, in articles and in discussions before societies; in fact, wherever the professional mind seeks an outlet for ideas, it apparently has the unfortunate tendency to neglect detailed examination of the history connected with the subject. This frequently is a source of intense mortification to the younger writers, when, after discussing some scientific problem, in which neglect is apparent in giving due acknowledgment to the labor of previous investigators, they are called to account for careless writing by some one more conversant with the facts in dental literature. The first duty of a writer, in assuming to elucidate some obscure problem, is to seek first the literature of the subject. This, in dentistry, is not a far search; perhaps a century and a half might cover it in its entirety, and possibly a half-century the period of greatest activity. When this has been completed the research worker is prepared to enter the arena of disputation fully armed for the conflict. The importance of this care in writing cannot be too frequently or too earnestly impressed on the younger generation of workers, who are enriching the literature of dentistry to-day more than at any previous period of its history. To be correct means labor, but it is a labor that is accompanied with its own great reward.

The editors of our journals have, it is presumed, more trouble with the careless habit of writers in giving proper names without examination than with all other errors combined. A name should never be written without careful verification. The average essayist apparently regards the editor, compositor, and proof-reader as the final arbiters of these difficult matters, evidently forgetting that their neglect very frequently means a serious loss of time for some one to make a lengthy search in order to be exact, for no journal of reputation can afford to exhibit ignorance in this direction. Unless the writer is absolutely assured that his spelling of the name is correct, he has no right to make use of it, to the possible injury of an original worker.

There is another and quite different exhibition of careless thinking, speaking, and writing, in the habit indulged in by some of ascribing to individuals, associations, and institutions statements, conclusions, and practices that do not belong to either. It is the loose method of setting up a man of straw to gain an opportunity to pull it to pieces without much labor. This sort of thing is manifested more frequently in charging dental educational institutions with all sorts of teachings not in harmony with the writer's conception of what constitutes a proper dental standard. The writer has been constantly impressed with this mental tendency in some otherwise very careful and capable writers. They will start out in an article on dental education, basing their conclusions on the knowledge of dental college work and dental college instruction obtained by them during their undergraduate experience ten, fifteen, or even forty years in the past. Upon this moss-grown experience they weave an article upon the curriculum of the present, and assume that the teaching of the dental colleges is not in accord with modern dental thought. The writer of this assumes that there does not exist a single mind capable of sounding the depth or the quality of the dental teaching in this country or any other in the range of civilization. This must be evident without waste of words in argument. The curricula of the various dental schools are given briefly in their annual announcements, but these are merely the skeletons, the soul, so to speak, being left to each individual teacher to fashion as he is prepared for the work. How impossible, then, is it for any outsider to declare that this is taught, or that is not taught, in the schools of dentistry anywhere. The curriculum of any dental college is, therefore, closed to all but the undergraduate of the immediate session in which he is a student. The teaching of each member of a faculty is practically a sealed book to every one of his colleagues. It must be, therefore, clear to all interested that knowledge of dental college teaching is an unwarranted assumption, even by those engaged intimately in the work. The writer would not attempt to express an opinion as to the value of any dental teaching unless he had an opportunity to examine the product. For this State boards of examiners have been provided, and we are obliged to be satisfied with this not very perfect solution of the problem.

The same careless method of thought and writing enters into all the several professional relations. Our moral law makes it a wrong to criticise a brother practitioner, but how many obey the code or hesitate to assume a more or less accurate knowledge of the practice of prominent dental practitioners. This would be amusing were it not a cause of much bad feeling, as it is an evidence of a lack of true professional courtesy. It is assuming to know our neighbor as we suppose we know ourselves, but, as we do not know ourselves, we are naturally ignorant of our neighbor's household.

The advice of the writer would be to those ambitious of entering the ranks of professional authors, to first acquire some degree of exactness in thought and then to make surety doubly sure that all the statements made will present an unassailable basis of truth. Style in writing may come by care and experience; good models may be of value, but they are not essential. Those who aim to acquire style in writing through this channel make a mistake with the result,—stilted composition. True style is individualized, and is an outgrowth of the spiritual nature developed through the refinement of general culture. Crude thinking is analogous to carelessness in the care of the body, for both may eventuate into a habit which, by use, is increasingly developed and is not, as a rule, ever eradicated, but becomes a bar to eventual success in all professional literary work.

